# Pediatric RSV-Associated Hospitalizations Before and During the COVID-19 Pandemic

**DOI:** 10.1001/jamanetworkopen.2023.36863

**Published:** 2023-10-04

**Authors:** Malou Bourdeau, Nirma Khatri Vadlamudi, Nathalie Bastien, Joanne Embree, Scott A. Halperin, Taj Jadavji, Kescha Kazmi, Joanne M. Langley, Marc H. Lebel, Nicole Le Saux, Dorothy Moore, Shaun K. Morris, Jeffrey M. Pernica, Joan Robinson, Manish Sadarangani, Julie A. Bettinger, Jesse Papenburg

**Affiliations:** 1 Department of Pediatrics, McGill University, Montreal, Quebec, Canada; 2 Department of Pediatrics, Faculty of Medicine, The University of British Columbia, Vancouver, British Columbia, Canada; 3 Vaccine Evaluation Center, BC Children’s Hospital Research Institute, University of British Columbia, Vancouver, British Columbia, Canada; 4 National Microbiology Laboratory, Public Health Agency of Canada, Winnipeg, Manitoba, Canada; 5 Department of Pediatrics, University of Manitoba, Winnipeg, Manitoba, Canada; 6 Canadian Center for Vaccinology, IWK Health Center, Dalhousie University, Halifax, Nova Scotia, Canada; 7 Section of Infectious Diseases, Department of Pediatrics, Alberta Children’s Hospital, University of Calgary, Calgary, Alberta, Canada; 8 Division of Infectious Diseases, Department of Pediatrics, Hospital for Sick Children, University of Toronto, Toronto, Ontario, Canada; 9 Division of Pediatric Infectious Diseases, Department of Pediatrics, Sainte-Justine, Montreal, Quebec, Canada; 10 Division of Infectious Diseases, Department of Pediatrics, Children’s Hospital of Eastern Ontario, Ottawa, Ontario, Canada; 11 Department of Pediatrics, McGill University, Montreal, Quebec, Canada; 12 Division of Infectious Diseases, Department of Pediatrics, McMaster University, Hamilton, Ontario, Canada; 13 Department of Pediatrics, University of Alberta, Edmonton, Alberta, Canada; 14 Department of Epidemiology, Biostatistics and Occupational Health, School of Population and Global Health, McGill University, Montreal, Quebec, Canada; 15 Division of Pediatric Infectious Diseases, Department of Pediatrics, Montreal Children’s Hospital, McGill University Health Centre, Montreal, Quebec, Canada; 16 Division of Microbiology, Department of Clinical Laboratory Medicine, McGill University Health Centre, Montreal, Quebec, Canada

## Abstract

**Importance:**

Respiratory syncytial virus (RSV) is a leading cause of pediatric hospitalizations.

**Objective:**

To describe the epidemiology and burden of RSV-associated hospitalizations among children and adolescents in Canadian tertiary pediatric hospitals from 2017 to 2022, including changes during the COVID-19 pandemic.

**Design, Setting, and Participants:**

This cross-sectional study was conducted during 5 RSV seasons (2017-2018 to 2021-2022) at 13 pediatric tertiary care centers from the Canadian Immunization Monitoring Program Active (IMPACT) program. Hospitalized children and adolescents aged 0 to 16 years with laboratory-confirmed RSV infection were included.

**Main Outcomes and Measures:**

The proportion of all-cause admissions associated with RSV and counts and proportions of RSV hospitalizations with intensive care unit (ICU) admission, prolonged stay (≥7 days), and in-hospital mortality were calculated overall and by season, age group, and region. Seasonality was described using epidemic curves. RSV hospitalizations for 2021-2022 were compared with those in the prepandemic period of 2017-2018 through 2019-2020. Bonferroni corrections were applied to *P* values to adjust for multiple statistical comparisons.

**Results:**

Among 11 014 RSV-associated hospitalizations in children and adolescents (6035 hospitalizations among male patients [54.8%]; 5488 hospitalizations among patients aged <6 months [49.8%]), 2594 hospitalizations (23.6%) had admission to the ICU, of which 1576 hospitalizations (60.8%) were among children aged less than 6 months. The median (IQR) hospital stay was 4 (2-6) days. The mean (SD) number of RSV-associated hospitalizations during prepandemic seasons was 2522 (88.8) hospitalizations. There were 58 hospitalizations reported in 2020-2021, followed by 3170 hospitalizations in 2021-2022. The proportion of all-cause hospitalizations associated with RSV increased from a mean of 3.2% (95% CI, 3.1%-3.3%) before the pandemic to 4.5% (95% CI, 4.3%-4.6%) in 2021-2022 (difference, 1.3 percentage points; 95% CI, 1.1-1.5 percentage points; corrected *P* < .001). A significant increase in RSV-associated hospitalizations was found in 2021-2022 for 3 provinces (difference range, 2.5 percentage points; 95% CI, 1.4-3.6 percentage points for Quebec to 2.9 percentage points; 95% CI, 1.4-3.5 percentage points for Alberta; all corrected *P* < .001). Age, sex, ICU admission, prolonged length of stay, and case fatality rate did not change in 2021-2022 compared with the prepandemic period. Interregional differences in RSV seasonality were accentuated in 2021-2022, with peaks for 1 province in October, 4 provinces in December, and 3 provinces in April, or May.

**Conclusions and Relevance:**

This study found that the burden of RSV-associated hospitalizations in Canadian pediatric hospitals was substantial, particularly among infants aged less than 6 months, and RSV hospitalizations increased in 2021-2022 compared with the prepandemic period, while severity of illness remained similar. These findings suggest that RSV preventive strategies for infants aged less than 6 months would be associated with decreased RSV disease burden in children.

## Key Points

**Question:**

What is the epidemiology of pediatric respiratory syncytial virus (RSV)–associated hospitalizations in Canada, and how has it changed with the COVID-19 pandemic?

**Findings:**

This cross-sectional study of 11 014 pediatric RSV-associated hospitalizations found that such hospitalizations in Canadian pediatric centers were frequent, particularly among children under age 6 months. After a near absence in 2020-2021, RSV admissions increased in 2021-2022 compared with prepandemic means, while severity and age distribution remained similar but with greater interregional variability in timing of RSV activity.

**Meaning:**

This study found a significant burden of RSV hospitalizations in Canadian pediatric centers, particularly among children under age 6 months. The study found an increase in admissions during 2021-2022, with regional variation in RSV activity timing.

## Teaser

This cross-sectional study investigates proportions of hospital admissions with RSV and rates of associated outcomes among children and adolescents before and during the COVID-19 pandemic.

## Introduction

Respiratory syncytial virus (RSV) is a leading cause of pediatric hospitalization worldwide, particularly in infants and young children. In North America and Europe, nearly 2% of all infants require hospitalization for RSV infection during the first year of life.^1,2^ Underlying health conditions, especially premature birth, chronic lung disease, and congenital heart disease, predispose children to severe RSV illness. Monthly immunoprophylaxis with palivizumab (Synagis, AstraZeneca) was until recently the only licensed measure to prevent severe RSV-associated lower respiratory tract infection in Canada, but its use was limited to infants at high risk and indications varied across Canadian provinces.^3,4^ However, healthy infants born at term account for most pediatric RSV hospitalizations.^5^ Long-acting monoclonal antibody prophylaxis that may be considered for all infants has recently become available in some jurisdictions, including in Canada and the US, and vaccines for use in pregnancy to protect young infants are currently under review.^6,7^

Public health measures put in place in March 2020 to counter SARS-CoV-2 transmission were associated with a marked reduction in respiratory illness attributed to bacteria and other viruses during the first year of the COVID-19 pandemic.^8,9^ In the Northern Hemisphere, an interseasonal surge in pediatric RSV–associated hospitalizations was observed in the summer and fall of 2021.^8^ To date, reports have been conflicting regarding changes in the age distribution and severity of RSV hospitalizations in children since the beginning of the COVID-19 pandemic,^10,11^ and national data from Canada, including possible interregional differences, are lacking. We aimed to describe overall, age-specific, and region-specific characteristics and in-hospital severity and outcomes of RSV-associated hospitalizations at Canadian Immunization Monitoring Program, Active (IMPACT) centers among children and adolescents aged 0 to 16 years during 5 RSV seasons (2017-2018 to 2021-2022) and to compare the 2021-2022 season with the prepandemic period.

## Methods

Institutional ethics approval was obtained at each IMPACT center for this cross-sectional study, with a waiver of informed consent due to the minimal risk of this health record review. This study follows the Strengthening the Reporting of Observational Studies in Epidemiology (STROBE) reporting guideline for observational studies.

### Study Design and Setting

We performed an active surveillance study of RSV-associated hospitalizations for 3 seasons (October 2017 to June 2018, November 2018 until June 2019, and October 2019 until June 2020) and year-round for 2 years (July 2020 until June 2022) among children and adolescents admitted to 12 tertiary care pediatric centers of the IMPACT network. Another pediatric center was added during July 2020 to June 2022; however, data from this site were excluded from all statistical comparisons between time periods. IMPACT hospitals account for approximately 90% of the pediatric tertiary care beds in Canada, with referrals from all provinces and territories.^12^

### Study Population

All children and adolescents aged 0 to 16 years who had laboratory-confirmed RSV infection, including hospital-acquired infection, at the time of admission or during their hospitalization were included. We could not distinguish between hospital-acquired and community-acquired infections.

### Data Collection

Trained nurse monitors screened daily laboratory results and admission lists for eligible RSV cases. Data were abstracted from medical records after patient discharge using a structured electronic case report form. Data entry forms contained drop-down menus and built-in logic to minimize errors and omissions. Data collected included hospital admission and discharge dates, age, sex, RSV specimen collection date, ICU admission, and disposition at discharge or transfer (died or survived). No data on underlying medical conditions were collected for this study. In addition, we captured the total number of all-cause medical inpatient admission days for each year from each IMPACT hospital (excluding psychiatry wards and newborn nurseries).

### Statistical Analysis

We considered 3 RSV seasons (2017-2018, 2018-2019, and 2019-2020) as the prepandemic period. Given the near absence of RSV laboratory detection in Canada and 58 IMPACT RSV cases during July 2020 to June 2021^13^ and considering the heterogeneity of pandemic health measures over time and across regions during that period, the 2020-2021 season was excluded from statistical comparisons. Data from the 2021-2022 season were compared with data from the 2017-2018 through 2019-2020 period using linear regression for continuous data (eg, age in months) and χ^2^ for categorical data (eg, sex and age group). *P* values ≤ .05 were considered statistically significant, and all comparisons were 2-sided. Overall and province-specific proportions of RSV-associated hospitalizations over all-cause hospitalizations were estimated. Individual proportions with 95% CIs were computed for overall and province specific proportions of RSV-associated hospitalizations over all-cause hospitalizations using prop.test in R statistical software. Changes in the proportion of RSV-associated hospitalizations were compared between means of the prepandemic period (2017-2018 to 2019-2020) and 2021-2022 using the 2 independent groups proportion test. Subgroup analyses were conducted to study disease severity differences by age group (ie, 0-5 months, 6-11 months, 12-23 months, 2-4 years, 5-9 years, and 10-16 years) using the 2 independent groups proportion test. Statistical analyses were performed using R statistical software version 4.1.3 (R Project for Statistical Computing). Bonferroni corrections were applied to *P* values to adjust for multiple statistical comparisons using the p.adjust function within the R stats package version 3.6.2. We reported individual level frequencies between 1 and 4 as less than 5 in accordance with IMPACT privacy policies.

## Results

### Population

**Figure 1.**
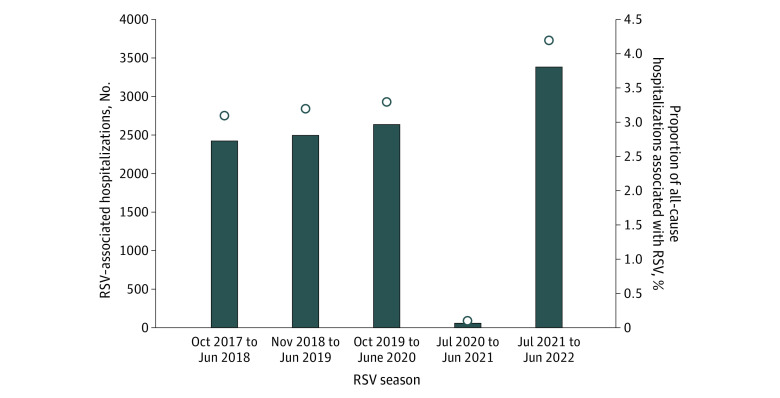
Frequency and Proportion of Respiratory Syncytial Virus (RSV) Hospitalizations Frequency and proportion of RSV hospitalizations over all-cause hospitalizations are presented by season at Immunization Monitoring Program Active (IMPACT) hospitals from 2017-2018 through 2021-2022. Bars indicate frequency of RSV-associated hospitalizations; open circles, proportion of RSV-associated hospitalizations over all-cause hospitalizations at IMPACT centers.

During the 5-year surveillance period, 11 014 RSV-associated hospitalizations (6035 hospitalizations among male patients [54.8%]; 5488 hospitalizations among children aged <6 months [49.8%] and 4163 hospitalizations among children aged 0-2 months [37.8%]) (eFigure 1 in Supplement 1) were reported across 13 IMPACT hospitals (Figure 1). Frequency peaked in the second month of life (1747 hospitalizations [15.9%]). The frequency of RSV hospitalization decreased with older age (eFigure 1 in Supplement 1). Age and sex distributions were similar during the prepandemic period (mean [SD] age, 1.4 [2.3] years; 4140 of 7568 hospitalizations among male patients [54.7%]) and 2021-2022 season (mean [SD] age, 1.3 [2.1] years; 1860 of 3388 hospitalizations among male patients [54.9%]) (Table 1).

**Table 1.** Age and Sex Distribution of RSV-Associated Hospitalizations

Characteristic	Hospitalizations with RSV infection, No. (%)						*P* value^b^
	2017-2018 (n = 2427)	2018-2019 (n = 2500)	2019-2020 (n = 2641)	2020-2021 (n = 58)	2021-2022 (n = 3388)	Total (N = 11 014)^a^	
Age, mo							
Mean (SD)	16.6 (28.6)	16.5 (28.3)	16.1 (25.9)	13.7 (26.1)	15.9 (25.4)	16.2 (26.9)	.33
Median (IQR)	6 (1-19)	6 (1-20)	6 (1-20)	4 (1-16)	5 (1-22)	6 (1-20)	
Sex							
Female	1105 (45.5)	1126 (45.0)	1196 (45.3)	23 (39.7)	1527 (45.1)	4977 (45.2)	.86
Male	1322 (54.5)	1373 (54.9)	1445 (54.7)	35 (60.3)	1860 (54.9)	6035 (54.8)	
Age group							
0-5 mo	1200 (49.4)	1241 (49.6)	1265 (47.9)	32 (55.2)	1750 (51.7)	5488 (49.8)	.76
6-11 mo	309 (12.7)	284 (11.4)	336 (12.7)	6 (10.3)	314 (9.3)	1249 (11.3)	.46
12-23 mo	416 (17.1)	443 (17.7)	471 (17.8)	12 (20.7)	546 (16.1)	1888 (17.1)	.49
2-4 y	293 (12.1)	337 (13.5)	365 (13.8)	5 (8.6)	519 (15.3)	1519 (13.8)	>.99
5-9 y	149 (6.1)	135 (5.4)	159 (6.0)	<5 (<8.6)	196 (5.8)	641 (5.8)	>.99
10-16 y	58 (2.4)	60 (2.4)	45 (1.7)	<5 (<8.6)	63 (1.9)	227 (2.1)	>.99

Abbreviation: RSV, respiratory syncytial virus.

^a^ Total number of infections for all 5 seasons (2017-2018 to 2021-2022).

^b^ The change in numeric variables, such as age during the pandemic season (2021-2022), was assessed using linear regression models compared with the prepandemic period (2017-2018 to 2019-2020). The change in categorical variables (sex and age group) was evaluated using χ^2^. The 2020-2021 period was excluded from all analyses in this table. Bonferroni corrected *P* values are presented for age groups.

### Yearly RSV Hospitalizations and Proportions of Admissions

The mean (range; SD) annual number of hospitalizations for the 3 prepandemic seasons (2017-2018 season to 2019-2020 season) was 2523 (2427-2641; 88.8) hospitalizations compared with a total of 3170 hospitalizations for the 2021-2022 season (Table 2). During the 2020-2021 season, 58 hospitalizations were reported and were limited geographically to 3 provinces, with 51 hospitalizations in the province of Quebec. Nationally, proportions of RSV hospitalizations over all-cause hospitalizations (Table 2) were a mean of 3.2% (95% CI, 3.1%-3.3%) during prepandemic seasons and were 4.5% (95% CI, 4.3%-4.6%) during the 2021-2022 season (difference, 1.3 percentage points; 95% CI, 1.1-1.5 percentage points; corrected *P* < .001). Changes in the proportion of RSV hospitalizations over all-cause hospitalizations in 2021-2022 were not consistent across regions; significant increases occurred in 3 provinces: Quebec (difference, 2.5 percentage points; 95% CI, 2.1-2.9 percentage points; corrected *P* < .001), Saskatchewan (difference, 2.5 percentage points; 95% CI, 1.4-3.6 percentage points; corrected *P* < .001), and Alberta (difference, 2.9 percentage points; 95% CI, 1.4-3.5 percentage points; corrected *P* < .001).

**Table 2.** Proportions of All-Cause Admissions With RSV Detected

Province	RSV admissions, No. (%^a^) [95% CI]						Difference (95% CI), percentage points^c^	*P* value^d^
	2017-2018	2018-2019	2019-2020	Prepandemic mean	2021-2022	Total^b^		
AB	533 (3.6) [3.3 to 3.9]	429 (2.9) [2.6 to 3.1]	522 (3.4) [3.2 to 3.7]	495 (3.3) [3.0 to 3.6]	831 (6.2) [5.8 to 6.7]	2317 (3.3) [3.0 to 3.4]	2.9 (2.4 to 3.5)	<.001
BC	184 (2.8) [2.4 to 3.2]	173 (2.9) [2.5 to 3.3]	150 (2.4) [2.0 to 2.8]	169 (2.7) [2.3 to 3.1]	182 (3.0) [2.6 to 3.5]	689 (2.3) [2.1 to 2.4]	0.3 (−0.3 to 1)	>.99
MB	187 (4.7) [4 to 5.4]	224 (5.3) [4.7 to 6.1]	252 (4.9) [4.4 to 5.6]	221 (5.0) [4.4 to 5.7]	182 (4.2) [3.6 to 4.8]	850 (3.9) [3.6 to 4.2]	−0.8 (−1.7 to 0.1)	.81
NL	59 (3.3) [2.5 to 4.2]	67 (3.6) [2.8 to 4.6]	68 (3.7) [2.9 to 4.7]	65 (3.6) [2.8 to 4.5]	24 (1.8) [1.2 to 2.7]	218 (2.7) [2.4 to 3.1]	−1.8 (−2.9 to −0.6)	.05
NS	121 (3.2) [2.6 to 3.8]	153 (4) [3.4 to 4.7]	179 (4.8) [4.1 to 5.5]	151 (4.0) [3.4 to 4.7]	132 (4.4) [3.7 to 5.2]	585 (3.4) [3.1 to 3.6]	0.4 (−0.6 to 1.4)	>.99
ON^e^	431 (1.9) [1.8 to 2.1]	517 (2.3) [2.1 to 2.5]	483 (2.2) [2 to 2.4]	477 (2.1) [2.0 to 2.3]	355 (1.8) [1.7 to 2.0]	1786 (1.7) [1.6 to 1.8 ]	−0.3 (−0.6 to 0)	.26
QC	809 (3.7) [3.4 to 3.9]	800 (3.6) [3.4 to 3.9]	847 (3.9) [3.6 to 4.2]	819 (3.7) [3.5 to 4.0]	1270 (6.2) [5.9 to 6.6]	3777 (3.6) [3.5 to 3.7]	2.5 (2.1 to 2.9)	<.001
SK	103 (2.9) [2.4 to 3.5]	137 (3.9) [3.3 to 4.6]	140 (4.1) [3.5 to 4.9 ]	127 (3.6) [3.0 to 4.3]	194 (6.1) [5.3 to 7.0]	574 (3.5) [3.2 to 3.8]	2.5 (1.4 to 3.6)	<.001
Overall	2427 (3.1) [3.0 to 3.2]	2500 (3.2) [3.1 to 3.3]	2641 (3.3) [3.2 to 3.4]	2523 (3.2) [3.1 to 3.3]	3170 (4.5) [4.3 to 4.6]	10 796 (2.9) [2.8 to 2.9]	1.3 (1.1 to 1.5)	<.001

Abbreviation: RSV, respiratory syncytial virus.

^a^ Percentages are calculated among total all-cause hospitalizations in Immunization Monitoring Program Active (IMPACT) centers of the province.

^b^ Total number of admissions with RSV for all 5 seasons (2017-2018 to 2021-2022).

^c^ The percentage point difference was calculated between the 2021-2022 season and the mean of the 3 prepandemic seasons (2017-2018 to 2019-2020); the 2020-2021 season was excluded from this analysis.

^d^ Bonferroni corrected *P* value.

^e^ An additional Ontario site participated as of July 1, 2020. Data from this site were excluded from this analysis.

### Disease Severity and Health Care Resource Use

Overall, 2594 hospitalizations (23.6%) had admission to the ICU (eTable in Supplement 1). Among admissions requiring ICU care, 590 admissions (22.7%) were first admitted to a ward and 2004 admissions (77.2%) were admitted directly to the ICU. Proportions of ICU admissions varied significantly across age groups (range, 214 of 1249 admissions among patients aged 6-11 months [17.1%] to 58 of 188 admissions among patients aged 10-16 years [30.9%]; *P* < .001).

More than one-quarter of hospitalizations among children aged younger than 6 months (1576 hospitalizations [28.7%]) had admission to the ICU, representing 60.8% of all ICU RSV admissions. RSV ICU admission proportion declined with age during the first year of life, from 537 of 1456 admissions (36.9%) among children in the first month to 26 of 163 admissions (16.0%) among children in the twelfth month (eFigure 2 in Supplement 1). The mean (SD) hospital length of stay (LOS) was 5.9 (17.5) days, with a median (IQR) of 4 (2-6) days, and 2347 hospitalizations (21.3%) had a LOS of more than 7 days. Moreover, mean (SD) LOS in patients admitted to the ICU (9.7 [18.5] days) was longer than that for patients not admitted to the ICU (4.7 [13.2] days; difference, 4.90 days; 95% CI, 4.13-5.67 days; *P* < .001). Overall, increasing age in months was associated with longer LOS (increase in LOS/1-mo increase in age = 0.02 days; 95% CI, 0.01-0.04 days; *P* < .001). Same-day discharges were uncommon, occurring in less than 3% of all age groups (highest proportion, 42 of 1770 hospitalizations among patients aged 2-4 years [2.4%]). Of a total of 29 in-hospital deaths (0.3% of admissions), 6 deaths (20.7%) occurred in children aged younger than 6 months.

Overall, proportions of RSV admissions with same-day discharge, ICU admission, prolonged LOS (≥7 days), and in-hospital mortality did not differ in the prepandemic period vs the 2021-2022 season. The differences were 0.8 percentage points (95% CI, 0.1 to 1.5 percentage points; *P* = .81) for same-day discharge, 0.2 percentage points (95% CI, −2.0 to 2.5; *P* > .99) for ICU admissions, −2.3 percentage points (95% CI, −4.5 to −0.2 percentage points; *P* = .90) for prolonged LOS, and 0.1 percentage points (95% CI, −0.2 to 0.4 percentage points; *P* > .99) for deaths (Table 3).

**Table 3.** Disease Severity of RSV-Associated Hospitalizations

Severity outcome	RSV hospitalizations, No. (%^a^) [95% CI]		Difference (95% CI), percentage points^b^	*P* value^c^
	Prepandemic mean (n = 2522)	2021-2022 (n = 3338)		
Same-day discharge	33 (1.3) [0.9 to 1.9]	71 (2.1) [1.7 to 2.7]	0.8 (0.1 to 1.5)	.81
ICU admission	592 (23.5) [21.8 to 25.2]	804 (23.7) [22.3 to 25.2]	0.2 (−2.0 to 2.5)	>.99
LOS ≥7 d	556 (22.0) [20.4 to 23.7]	668 (19.7) [18.4 to 21.1]	−2.3 (−4.5 to −0.2)	.90
Death	6 (0.2) [0.1 to 0.5]	11 (0.3) [0.2 to 0.6]	0.1 (−0.2 to 0.4)	>.99

Abbreviations: ICU, intensive care unit; LOS, length of stay; RSV, respiratory syncytial virus.

^a^ Percentages are calculated among total admissions with RSV for that time period.

^b^ The percentage point difference was calculated between the 2021-2022 season and the mean of the 3 prepandemic seasons (2017-2018 to 2019-2020); the 2020-2021 season was excluded from this analysis. An additional Ontario site participated as of July 1, 2020, but data from this site were excluded from the statistical comparisons.

^c^ Bonferroni corrected *P* value.

### Geographic Distribution and Seasonality

**Figure 2.**
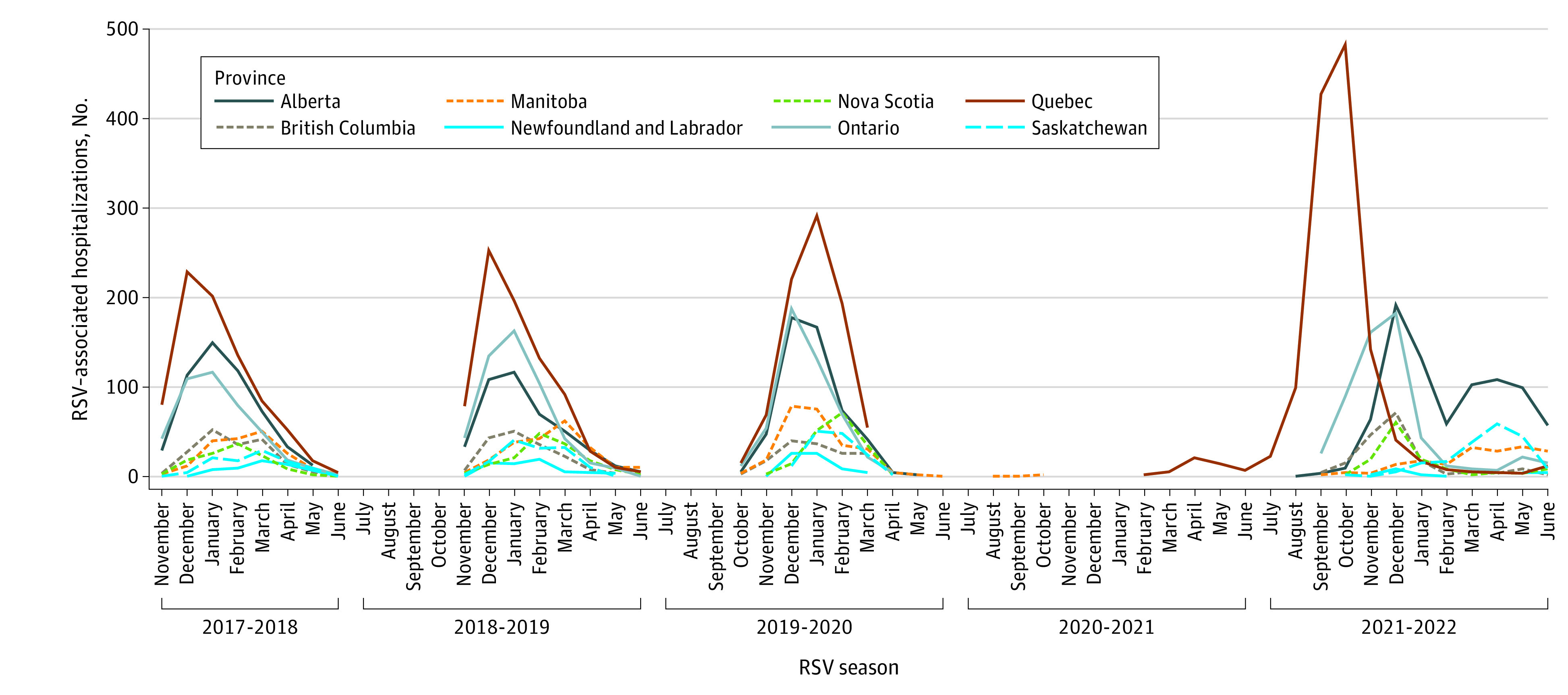
Monthly Respiratory Syncytial Virus (RSV)–Associated Hospitalizations Monthly hospitalizations at Immunization Monitoring Program Active (IMPACT) hospitals, 2017-2018 through 2021-2022, are presented by province and year.

From 2017-2018 through 2019-2020, national weekly RSV hospitalization counts peaked in January or February (eFigure 3 in Supplement 1) and monthly counts peaked in January (Figure 2). In 2021-2022, there was a bimodal distribution nationally, with weekly peaks occurring in October and December. Interregional differences in monthly peak hospitalizations were accentuated in the 2021-2022 season, with peaks for 1 province in October, 4 provinces in December, and 3 provinces in April or May (Figure 2).

## Discussion

In this national active-surveillance cross-sectional study, we described the epidemiology of RSV hospitalizations at pediatric hospitals in Canada during 3 RSV seasons before and 2 seasons after the onset of the COVID-19 pandemic. The hospitalization burden of RSV in Canadian pediatric centers was considerable, with a mean of more than 2500 RSV admissions annually, excluding the 2020-2021 season. The highest burden was seen in young infants, with half of RSV admissions occurring in the first 6 months of life and nearly 40% in the ages 0 to 2 months group.

RSV-associated ICU admissions were frequent, occurring in nearly one-quarter of children and adolescents with RSV. Infants aged 0 to 5 months accounted for 60.8% of all RSV ICU admissions. A comparable proportion of children requiring ICU care (17.4%) were seen in a study of tertiary pediatric centers in the US.^14^ However, data from tertiary care centers are biased toward patients with severe illness and are not generally representative of the broader population of children and adolescents hospitalized with RSV. A population-based study^15^ in Ontario, Canada, estimated the ICU admission proportion across all pediatric RSV hospitalizations in children aged younger than 3 years to be 5.6%.

Overall, in-hospital mortality occurred in 0.3% of RSV hospitalizations, which is comparable to the reported range of 0.04% to 0.90% in large US studies.^16^ Our data did not allow us to ascertain if RSV contributed to death. Thus, our in-hospital mortality rate is potentially an overestimate of RSV-attributable mortality. A Canadian study^17^ of RSV mortality from 11 pediatric centers during 2003 to 2013 identified 90 deaths during RSV admissions. Death was completely unrelated to the RSV infection in 12% of deaths and due to RSV in 36% of deaths, while the virus was a contributor in 34% of deaths and played an undetermined role in 20% of deaths.

The near absence of pediatric RSV hospitalizations in Canada during the first year of the COVID-19 pandemic was commensurate with exceptionally low levels of circulation of RSV and other respiratory viruses nationally, similar to observations in other countries.^9,10,18,19^ This was most likely associated with the implementation of COVID-19 pandemic public health measures, such as school and daycare closures, masking, travel restrictions, and physical distancing.^8^ Similarly, no pediatric influenza–associated hospitalizations or deaths were reported at IMPACT centers during the 2020-2021 season.^20^

We observed an overall increase in pediatric RSV–associated hospitalizations in Canada after the first year of the COVID-19 pandemic, beginning in the summer of 2021. However, this increase was primarily associated with higher RSV admission rates observed in 3 provinces; other regions had no significant change in the proportion of RSV-associated admissions in children and adolescents. This resurgence of RSV infections was likely associated with reduced population-level immunity caused by the absence of infections in the previous year, combined with children’s reengagement in social activities and the relaxing of other COVID-19 public health measures.^21^ Other countries observed similar increases in RSV-associated disease, with some reports noting that it occurred without the expected proportional increases in infections and hospitalizations in older adults.^10,22,23,24^ The age distribution of children and adolescents hospitalized with RSV at IMPACT sites remained similar in 2021-2022 compared with pre-COVID-19 seasons, with approximately half of all patients younger than age 6 months. Some studies found an increase in the proportion of older children testing positive or admitted with RSV.^25,26^ In British Colombia, the median age of children aged less than 36 months testing positive for RSV increased from 6.3 months to 11.8 months in the 2021-2022 season^27^; however, no change in age distribution among hospitalized patients was observed. It has been hypothesized that when pandemic restrictions were loosened, the absence of circulating RSV during the 2020-2021 winter season resulted in a cohort of older infants who were RSV-naive and more susceptible to lower respiratory tract RSV infections; these illnesses required medical attention but were not severe enough to require hospital admission given that hospitalization risk decreases with age.^2,15^ We did not observe an increase in the age of children and adolescents at RSV admission or an increase in disease severity (ICU admission, prolonged LOS [≥7 days], or mortality) overall or within any age stratum in 2021-2022. However, a national, population-based study^10^ in Denmark in 2021-2022 found an increase in the incidence rate of RSV-associated admissions and mechanical ventilation, especially among children aged 2 to 5 years, underscoring the importance of ongoing monitoring of age-specific disease incidence of RSV.

Given that our observed age distribution of pediatric RSV disease was heavily weighted toward young infants, preventive strategies, such as long-acting monoclonal antibody immunoprophylaxis for all infants and RSV vaccines for pregnant persons, could be associated with profound decreases in RSV burden. The efficacy of a single dose of the monoclonal antibody nirsevimab (Sanofi and AstraZeneca) has been estimated to be 77% against RSV-associated hospital admissions through 150 days after the dose in healthy infants born at full term or before term (≥29 weeks’ gestational age).^28,29^ Efficacy estimates of the RSV bivalent PreF vaccine in pregnancy (Pfizer) for severe medically attended RSV lower respiratory tract infection in infants are 82% through the first 90 days of life and 69% through 6 months.^30^

Monitoring RSV seasonality is essential for pediatric health care capacity planning, including the appropriate timing of monthly palivizumab immunoprophylaxis, generally administered from November to March in Canada. During the 2021-2022 season, there was increased variability in seasonality between provinces, with some regions experiencing early peaks of RSV activity during the summer and fall of 2022, and typically small interregional differences were greatly accentuated. In Quebec, a summer 2021 surge in RSV admissions and an abrupt halt in transmission in January 2022 prompted a start and end of the provincial palivizumab program approximately 2 months earlier than planned. Prairie provinces, such as Manitoba, Saskatchewan, and Alberta, had to extend their palivizumab programs due to ongoing RSV admissions in the spring of 2022 by contrast. This highlights the importance of regional surveillance for RSV, especially considering that optimizing the cost-effectiveness of long-acting monoclonal antibodies and maternal vaccination against RSV will depend in part on the timing of their administration in relation to RSV activity.^31^

### Limitations

Our study has limitations. Surveillance was restricted to tertiary care centers in Canada; therefore, results may not be generalizable to the entirety of Canadian RSV pediatric hospitalizations. Changes in RSV testing practices likely occurred over time, particularly after the onset of the COVID-19 pandemic, and such changes may have varied across IMPACT centers, affecting case capture. It is possible that diagnostic biases may have led to an overestimation of the increase in RSV hospitalizations in 2021-2022 or an underestimation of the disease burden in older children and adolescents, who less typically experience severe RSV. Thresholds for admission and health-seeking behaviors may also have been affected during this study period due to health care system pressures related to the pandemic. Due to the limited number of variables collected, we could not identify incidental RSV infections unrelated to the reason for admission or distinguish hospital-acquired infections. Moreover, we were unable to determine the underlying medical conditions of hospitalized children. There were also gaps in the surveillance period during the first 3 seasons, in which data were not collected during summer months. However, national laboratory surveillance for RSV did not suggest significant circulation during these periods, indicating that few if any cases would have been missed.^9^ Despite these limitations, this study represents nearly 90% of pediatric tertiary care beds in Canada, providing a comprehensive view of RSV hospitalizations over a 5-year period and allowing for comparisons over time and across geographic areas.

## Conclusions

This cross-sectional study found that RSV hospitalizations were frequent in Canadian pediatric centers and that nearly 1 in 4 hospitalized children and adolescents required ICU care. The greatest burden was among children aged less than 6 months, representing approximately half of RSV hospitalizations and 60% of RSV ICU admissions. After a near absence of RSV admissions during the winter of 2020-2021, there was an overall increase in pediatric RSV admissions in 2021-2022, with regional variation. There were no changes over time in age distribution or the proportion of patients requiring ICU admission or hospital admission of 7 days or longer; however, there was greater interregional variability in the timing of RSV activity in 2021-2022. Taken together, our results suggest that RSV preventive strategies for infants aged less than 6 months may have the greatest potential for associated decreases in RSV burden in children and adolescents. Our findings also highlight the importance of ongoing surveillance of pediatric RSV hospitalizations at national and regional levels.

## Notes

Supplement 1. **eFigure 1.** Proportion of Respiratory Syncytial Virus–Associated Hospitalizations by Age **eFigure 2.** Intensive Care Unit Admission by Age in Infants Aged <1 y **eFigure 3.** Weekly Respiratory Syncytial Virus–Associated Hospital Admissions by Season **eTable.** Disease Severity by Age Group

Supplement 2. **Nonauthor Collaborators.** Canadian Immunization Monitoring Program Active (IMPACT) Investigators

Supplement 3.
Data Sharing Statement
