# Supplementary material for: Pediatric RSV-Associated Hospitalizations Before and During the COVID-19 Pandemic
Source: JAMA Netw Open. 2023 Oct 4;6(10):e2336863. doi: 10.1001/jamanetworkopen.2023.36863 (PMC10551765; doi:10.1001/jamanetworkopen.2023.36863)
Supplement: Supplement 1. — eFigure 1. Proportion of Respiratory Syncytial Virus–Associated Hospitalizations by Age eFigure 2. Intensive Care Unit Admission by Age in Infants Aged <1 y eFigure 3. Weekly Respiratory Syncytial Virus–Associated Hospital Admissions by Season eTable. Disease Severity by Age Group [file jamanetwopen-e2336863-s001.pdf]

Supplemental Online Content

Bourdeau M, Vadlamudi NK, Bastien N, et al; for the Canadian Immunization Monitoring Program Active (IMPACT) Investigators. Pediatric RSV-associated hospitalizations before and during the COVID-19 pandemic. *JAMA Netw Open*. 2023;6(10):e2336863.
doi:10.1001/jamanetworkopen.2023.36863

eFigure 1. Proportion of Respiratory Syncytial Virus–Associated Hospitalizations by Age
eFigure 2. Intensive Care Unit Admission by Age in Infants Aged <1 y
eFigure 3. Weekly Respiratory Syncytial Virus–Associated Hospital Admissions by Season
eTable. Disease Severity by Age Group

This supplemental material has been provided by the authors to give readers additional information about their work.

© 2023 Bourdeau M et al. *JAMA Network Open.*

eFigure 1. Proportion of respiratory syncytial virus-associated hospitalizations by age, 2017-2018 to 2021-2022.

A. RSV-associated hospitalizations as a proportion of all hospitalizations by month of life in children < 1 year old

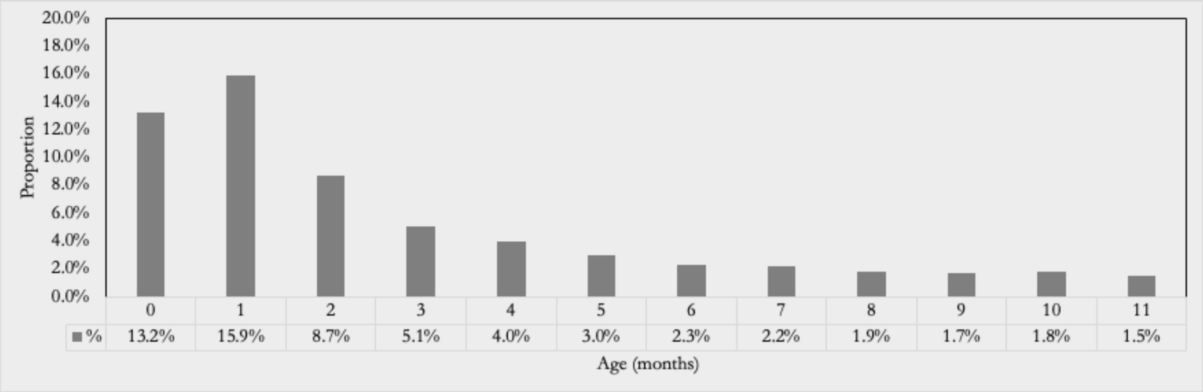

B. RSV-associated hospitalizations as a proportion of all hospitalizations by year of life

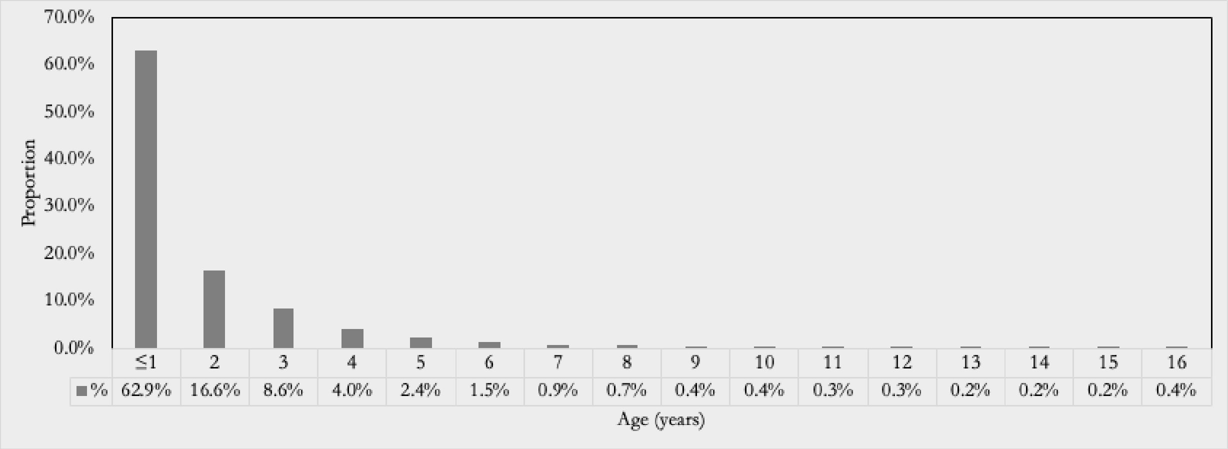

© 2023 Bourdeau M et al. *JAMA Network Open.*

eFigure 2: Intensive care unit admission by age in infants aged <1 year, 2017-2018 to 2021-2022

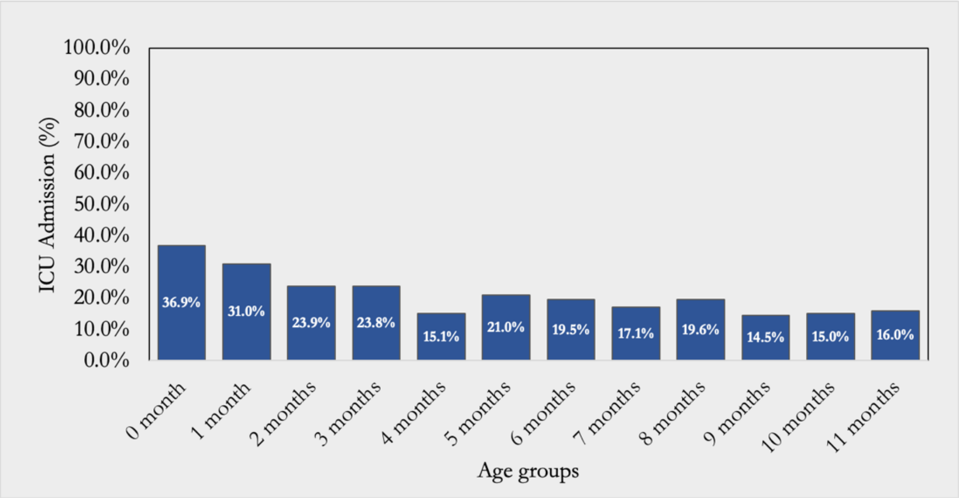

We present the proportion of infants admitted to ICU compared with all those admitted associated with RSV hospitalization by one-month age band. For example, 1456 infants were admitted to IMPACT hospital with RSV during the first month of life. Of these, 537 (36.9%) were admitted to ICU.

© 2023 Bourdeau M et al. *JAMA Network Open.*

eFigure 3: Weekly respiratory syncytial virus-associated hospital admissions, by season, 2017-2018 to 2021-2022

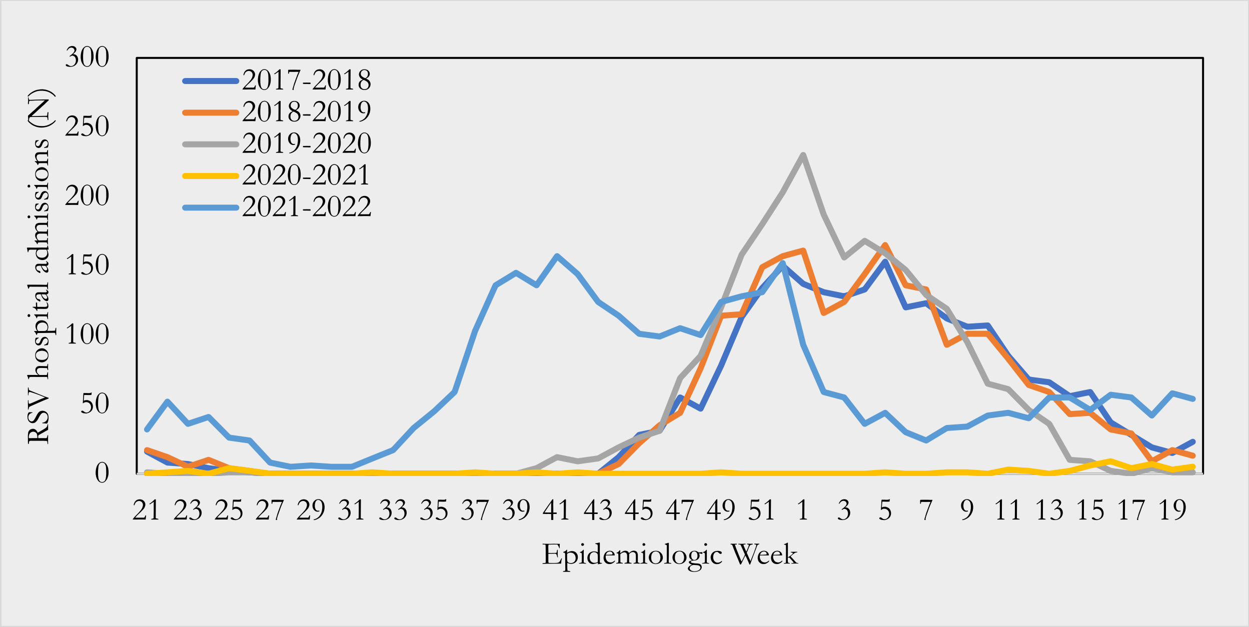

© 2023 Bourdeau M et al. *JAMA Network Open.*

eTable: Disease severity by age group

	Pre-pandemic mean, No. (%; 95% CI)	2021-2022, No. (%; 95% CI)	Total, No. (%; 95% CI)l[a]	Difference (95% CI), percentage points[b]	P-value[c]
**Overall**	**(N=2523)**	**(N=3388)**	**(N=11014)**		
Same day discharge	33 (1.3 ; 0.9-1.9)	71 (2.1 ; 1.7-2.7)	175 (1.6 ; 1.4-1.8)	0.8 (0.1-1.5)	0.81
ICU Admission	592 (23.5 ; 21.8-25.2)	804 (23.7 ; 22.3-25.2)	2594 (23.6 ; 22.8-24.4)	0.2 (-2-2.5)	1.00
LOS ≥ 7 days	556 (22 ; 20.4-23.7)	668 (19.7 ; 18.4-21.1)	2347 (21.3 ; 20.6-22.1)	-2.3 (-4.5--0.2)	0.90
Death	6 (0.2 ; 0.1-0.5)	11 (0.3 ; 0.2-0.6)	29 (0.3 ; 0.2-0.4)	0.1 (-0.2-0.4)	1.00
**0--5 months**	**(N=1235)**	**(N=1750)**	**(N=5488)**		
Same day discharge	13 (1.1 ; 0.6-1.8)	27 (1.5 ; 1-2.3)	69 (1.3 ; 1-1.6)	0.4 (-0.4-1.4)	1.00
ICU Admission	358 (29 ; 26.5-31.6)	489 (27.9 ; 25.9-30.1)	1576 (28.7 ; 27.5-29.9)	-1.1 (-4.4-2.3)	1.00
LOS ≥ 7 days	281 (22.8 ; 20.5-25.2)	379 (21.7 ; 19.8-23.7)	1228 (22.4 ; 21.3-23.5)	-1.1 (-4.2-2)	1.00
Death	<5	<5	6 (0.1 ; 0-0.3)	…	…
**6-11 months**	**(N=310)**	**(N=314)**	**(N=1249)**		
Same day discharge	<5	10 (3.2 ; 1.6-6)	18 (1.4 ; 0.9-2.3)	2.2 (-0.3-4.8)	1.00
ICU Admission	53 (17.1 ; 13.2-21.9)	55 (17.5 ; 13.6-22.3)	214 (17.1 ; 15.1-19.4)	0.4 (-5.8-6.7)	1.00
LOS ≥ 7 days	59 (19 ; 14.9-23.9)	51 (16.2 ; 12.4-20.9)	228 (18.3 ; 16.2-20.5)	-2.8 (-9.1-3.5)	1.00
Death	<5	<5	<5	…	…
**12-23 months**	**(N=443)**	**(N=546)**	**(N=1888)**		
Same day discharge	7 (1.6 ; 0.7-3.4)	14 (2.6 ; 1.5-4.4)	36 (1.9 ; 1.4-2.7)	1 (-1-3)	1.00
ICU Admission	77 (17.4 ; 14-21.3)	99 (18.1 ; 15-21.7)	329 (17.4 ; 15.8-19.2)	0.7 (-4.2-5.7)	1.00
LOS ≥ 7 days	72 (16.3 ; 13-20.1)	76 (13.9 ; 11.2-17.2)	294 (15.6 ; 14-17.3)	-2.4 (-7-2.4)	1.00
Death	<5	<5	7 (0.4 ; 0.2-0.8)	…	…
**2-4 years**	**(N=390)**	**(N=593)**	**(N=1770)**		
Same day discharge	8 (2.1 ; 1-4.2)	17 (2.9 ; 1.7-4.6)	42 (2.4 ; 1.7-3.2)	0.8 (-1.3-3)	1.00
ICU Admission	64 (16.4 ; 12.9-20.5)	111 (18.7 ; 15.7-22.1)	304 (17.2 ; 15.5-19)	2.3 (-2.7-7.4)	1.00
LOS ≥ 7 days	85 (21.8 ; 17.9-26.3)	96 (16.2 ; 13.4-19.5)	354 (20 ; 18.2-22)	-5.6 (-10.9--0.3)	0.92
Death	<5	<5	7 (0.4 ; 0.2-0.9)	…	…
**5-9 years**	**(N=98)**	**(N=134)**	**(N=429)**		
Same day discharge	<5	<5	8 (1.9 ; 0.9-3.8)	-2 (-5.7-1.6)	1.00
ICU Admission	26 (26.5 ; 18.4-36.6)	33 (24.6 ; 17.8-33)	112 (26.1 ; 22.1-30.6)	-1.9 (-14.2-10.4)	1.00
LOS ≥ 7 days	38 (38.8 ; 29.3-49.2)	40 (29.9 ; 22.4-38.5)	155 (36.1 ; 31.6-40.9)	-8.9 (-22.2-4.3)	1.00
Death	<5	<5	6 (1.4%)	…	…
**10-16 years**	**(N=45)**	**(N=51)**	**(N=188)**		
Same day discharge	<5	<5	<5	…	…
ICU Admission	14 (31.1 ; 18.6-46.8)	17 (33.3 ; 21.1-48)	58 (30.9 ; 24.4-38.1)	2.2 (-18.6-23)	1.00
LOS ≥ 7 days	21 (46.7 ; 31.9-62)	26 (51 ; 36.8-65)	88 (46.8 ; 39.6-54.2)	4.3 (-17.8-26.4)	1.00
Death	<5	<5	<5	…	…

© 2023 Bourdeau M et al. *JAMA Network Open.*

N, count; %, calculated as count divided by total RSV cases for the age category for that time period times100; m, months; y, years; ICU, intensive care unit; LOS, length of stay; d, days; CI, confidence interval

[a]Total number of cases for all five seasons, 2017-2018 to 2021-2022.
[b]Percentage point difference between the 2021-2022 season and the average of the three pre-pandemic seasons, 2017-2018 to 2019-2020; 2020-2021 was excluded from this analysis; an additional Ontario site participated as of July 1, 2020-2022. Data from this site were excluded from the statistical comparisons
[c]Bonferroni corrected p-value

© 2023 Bourdeau M et al. *JAMA Network Open.*
