# Supplementary material for: Pediatric RSV-Associated Hospitalizations Before and During the COVID-19 Pandemic
Source: JAMA Netw Open. 2023 Oct 4;6(10):e2336863. doi: 10.1001/jamanetworkopen.2023.36863 (PMC10551765; doi:10.1001/jamanetworkopen.2023.36863)
Supplement: Supplement 2. — Nonauthor Collaborators. Canadian Immunization Monitoring Program Active (IMPACT) Investigators [file jamanetwopen-e2336863-s002.pdf]

*Group Name(s): Canadian Immunization Monitoring Program Active (IMPACT) Investigators							
***First Name and Middle Initial(s)**	***Last Name**	***Suffix (eg, Jr, III)**	Academic Degrees	Institution	Location (city, state/province, country)	Role or Contribution, eg, chair, principal investigator	Group (if more than 1 Group listed in the byline) and/or Subgroup (eg, Steering Committee)
Cheryl	Foo		MD	Janeway Children’s Health & Rehabilitation Centre	St. John’s, NL, Canada	Site investigator	
Natalie	Bridger		MD	Janeway Children’s Health & Rehabilitation Centre	St. John’s, NL, Canada	Site investigator	
Scott A.	Halperin		MD	IWK Health Centre	Halifax, NS, Canada	Principal investigator	
Karina A.	Top		MD	IWK Health Centre	Halifax, NS, Canada	Site investigator	
Roseline	Thibeault		MD	Centre Mere-Enfant de Quebec, CHUL	Quebec City, QC, Canada	Site investigator	
Dorothy	Moore		MD, PhD	Montreal Children’s Hospital, McGill University Health Centre	Montreal, QC, Canada	Site investigator	
Jesse	Papenburg		MD, MSc	Montreal Children’s Hospital, McGill University Health Centre	Montreal, QC, Canada	Site investigator	
Marc H.	Lebel		MD	Centre hospitalier universitaire Sainte-Justine	Montreal, QC, Canada	Site investigator	
Nicole	Le Saux		MD	Children’s Hospital of Eastern Ontario	Ottawa, ON, Canada	Site investigator	
Shaun K.	Morris		MD, MPH	Hospital for Sick Children	Toronto, ON, Canada	Site investigator	
Kescha	Kamzi		MD	Hospital for Sick Children	Toronto, ON, Canada	Site investigator	
Rupeena	Purewal		MD	Jim Pattison Children’s Hospital	Saskatoon, SK, Canada	Site investigator	
Rupesh	Chawla		MD	Jim Pattison Children’s Hospital	Saskatoon, SK, Canada	Site investigator	
Taj	Jadavji		MD	Alberta Children’s Hospital	Calgary, AB, Canada	Site investigator	
Catherine	Burton		MD	Stollery Children’s Hospital	Edmonton, AB, Canada	Site investigator	
Julie A.	Bettinger		MD, PhD	BC Children’s Hospital	Vancouver, BC, Canada	Lead epidemiologist	
Manish	Sadarangani		BM BCh, DPhil	BC Children’s Hospital	Vancouver, BC, Canada	Site investigator	
Laura	Sauvé		MD	BC Children’s Hospital	Vancouver, BC, Canada	Site investigator	
Jared	Bullard		MD	Winnipeg Children’s Hospital Health Sciences Center	Winnipeg, MN, Canada	Site investigator	
Joanne	Embree		MD	Winnipeg Children’s Hospital Health Sciences Center	Winnipeg, MN, Canada	Site investigator	
Jeffrey	Pernica		MD, MSc	McMaster Children Hospital	Hamilton, ON, Canada	Site investigator	
