# Supplementary material for: Pediatric RSV-Associated Hospitalizations Before and During the COVID-19 Pandemic
Source: JAMA Netw Open. 2023 Oct 4;6(10):e2336863. doi: 10.1001/jamanetworkopen.2023.36863 (PMC10551765; doi:10.1001/jamanetworkopen.2023.36863)
Supplement: Supplement 3. — Data Sharing Statement [file jamanetwopen-e2336863-s003.pdf]

Data Sharing Statement

Bourdeau. Pediatric RSV-Associated Hospitalizations Before and During the COVID-19 Pandemic. *JAMA Netw Open*. Published online October 4, 2023. doi:10.1001/jamanetworkopen.2023.36863

Data
Data available: No

Additional Information
Explanation for why data not available: The data are owned by the Public Health Agency of Canada
